# Real-World Guideline Concordance and Potential Overtreatment Following Primary Radical Hysterectomy for Early-Stage Cervical Cancer

**DOI:** 10.3390/jcm15207952

**Published:** 2026-10-14

**Authors:** Sinan Ateş, Arzu Ay, Gonca Büşra Çoker, Nisa Gjergjizi, Rona Luboteni, Füsun Varol, N. Cenk Sayın

**Affiliations:** 1 Department of Obstetrics and Gynecology, Faculty of Medicine, Trakya University, Edirne 22030, Turkey; nisagjergjizi@hotmail.com (N.G.); rronaluboteni@gmail.com (R.L.); fgvarol@yahoo.com (F.V.); ncsayin@yahoo.com (N.C.S.); 2 Department of Biophysics, Faculty of Medicine, Trakya University, Edirne 22030, Turkey; arzuay@trakya.edu.tr; 3 Uzunköprü State Hospital, Uzunköprü, Edirne 22200, Turkey; goncacoker@gmail.com

**Keywords:** early-stage cervical cancer, radical hysterectomy, adjuvant therapy, guideline concordance, ESGO/ESTRO/ESP guidelines, overtreatment, practice patterns, disease-free survival

## Abstract

**Background/Objectives:** International guidelines recommend risk-adapted postoperative management in early-stage cervical cancer; however, the extent to which these recommendations are implemented in routine clinical practice remains incompletely characterized, particularly among patients without high-risk pathological features. This study evaluated real-world concordance with the 2023 European Society of Gynecological Oncology/European Society for Radiotherapy and Oncology/European Society of Pathology (ESGO/ESTRO/ESP) recommendations and investigated the clinical implications of potential overtreatment. **Methods:** We retrospectively evaluated a single-center cohort of 135 patients treated with primary radical hysterectomy for early-stage cervical cancer at our institution. Postoperative pathological risk factors were used to stratify patients in accordance with the 2023 ESGO/ESTRO/ESP guidelines. Postoperative management was classified as guideline-concordant or potential overtreatment. Disease-free survival (DFS) and overall survival (OS) were assessed by Kaplan–Meier analysis and univariable Cox proportional hazards regression. **Results:** The high-risk group comprised 50 patients (37.0%), and the non-high-risk group comprised the remaining 85 patients (63.0%). Among non-high-risk patients, 28 (32.9%) received guideline-concordant postoperative management, whereas 57 (67.1%) received treatment classified as potential overtreatment. The distribution of postoperative treatment intensity differed significantly according to guideline-concordance status (*p* < 0.001). However, DFS did not differ significantly between the guideline-concordant and potential overtreatment groups (log-rank *p* = 0.62; hazard ratio [HR] = 1.38, 95% confidence interval [CI] 0.38–5.06), nor did OS (log-rank *p* = 0.85; HR = 1.09, 95% CI 0.42–2.84). **Conclusions:** Postoperative management showed substantial variation among patients without high-risk pathological features, with potential overtreatment observed in approximately two-thirds of this cohort. No statistically significant differences in DFS or OS were observed between the groups; however, the limited number of events and wide confidence intervals preclude exclusion of clinically meaningful treatment effects.

## 1. Introduction

Cervical cancer remains one of the most common gynecologic malignancies worldwide and continues to pose a major public health challenge, with approximately 662,000 new cases and 349,000 deaths estimated globally in 2022 [1]. Although organized screening and vaccination against human papillomavirus (HPV) have reduced the burden of cervical cancer in many high-income countries, it remains disproportionately high in low- and middle-income regions [1,2]. For patients with early-stage disease, primary radical hysterectomy accompanied by pelvic lymph node assessment remains one of the principal curative treatment strategies [3].

Following primary surgery, postoperative management is guided by pathological factors associated with recurrence risk [3]. Contemporary European Society of Gynecological Oncology/European Society for Radiotherapy and Oncology/European Society of Pathology (ESGO/ESTRO/ESP) and National Comprehensive Cancer Network (NCCN) guidelines recommend tailoring postoperative treatment to pathological risk, with specific risk features informing decisions among observation, radiotherapy, and concurrent chemoradiotherapy [3,4]. High-risk pathological features—including pelvic lymph-node metastasis, parametrial involvement, and positive surgical margins—are clear indications for adjuvant concurrent chemoradiotherapy [3,5]. In contrast, management of patients without high-risk pathological features, particularly those with intermediate-risk features, remains more nuanced because treatment decisions rely on combinations of pathological risk factors rather than a single definitive indication [3,6]. Although several studies have evaluated adherence to cervical cancer treatment guidelines, real-world evidence regarding postoperative guideline concordance and potential overtreatment remains limited [7,8,9].

Adherence to cervical cancer treatment guidelines varies across healthcare settings, with substantial differences in guideline-concordant management reported in population-based studies [7]. Recent studies indicate that postoperative management of intermediate-risk patients remains heterogeneous, with considerable variability in the use of adjuvant radiotherapy and concurrent chemoradiotherapy despite contemporary recommendations [10,11]. Treatment intensification is nevertheless common in this population. Recent population-based evidence indicates that concurrent chemotherapy is frequently added to postoperative radiotherapy despite the absence of a demonstrated survival benefit, raising concerns regarding potential overtreatment and avoidable treatment-related morbidity [11,12]. However, relatively few comparative studies have directly assessed the oncologic impact of escalating adjuvant treatment in intermediate-risk patients, and available findings remain heterogeneous [10,13]. Furthermore, existing studies have largely focused on treatment patterns or comparative treatment outcomes, whereas real-world guideline concordance, potential overtreatment, and oncologic outcomes have rarely been examined together within the same cohort using contemporary ESGO/ESTRO/ESP recommendations [9,10,13]. Consequently, further real-world evaluation of guideline implementation, treatment concordance, and associated clinical outcomes remains warranted [3,7,8].

Therefore, the present study aimed to assess the extent to which postoperative treatment in routine practice aligned with the 2023 ESGO/ESTRO/ESP recommendations among patients treated with primary radical hysterectomy for early-stage cervical cancer [3]. We further sought to quantify the frequency of potential overtreatment among patients without high-risk pathological features and to examine whether treatment intensification was related to disease-free survival and overall survival. By evaluating both guideline implementation and survival outcomes, this study provides contemporary real-world evidence regarding the extent and potential clinical implications of postoperative treatment intensification.

## 2. Materials and Methods

### 2.1. Study Design and Patient Population

We retrospectively evaluated a single-center cohort of patients with FIGO stage IA–IIB cervical cancer who underwent primary radical hysterectomy at Trakya University Faculty of Medicine between January 2012 and December 2025. Clinical, pathological, treatment, and follow-up data were obtained through retrospective review of institutional electronic medical records and hospital databases. Patients were excluded if clinicopathological variables required for postoperative risk stratification (deep stromal invasion, tumor size, lymph vascular space invasion, pelvic lymph-node status, parametrial involvement, or surgical margin status) were missing, postoperative treatment information was incomplete, or follow-up data required for disease-free survival (DFS) or overall survival (OS) analyses were unavailable. Consequently, the final analysis comprised 135 patients with complete datasets.

### 2.2. Postoperative Risk Stratification

Patients were initially categorized according to classical postoperative pathological risk factors. High-risk disease was assigned when at least one high-risk pathological feature was present—pelvic lymph-node metastasis, parametrial involvement, or positive surgical margins—consistent with established high-risk criteria and the 2023 ESGO/ESTRO/ESP recommendations [3,5]. Patients with none of these features formed the non-high-risk cohort.

Among non-high-risk patients, intermediate-risk pathological factors were subsequently assessed using a simplified Sedlis-type framework incorporating tumor diameter ≥ 4 cm, deep stromal invasion, and lymphovascular space invasion (LVSI) [6,7]. Patients with two or more of these three factors were considered intermediate-risk for the purpose of postoperative treatment assessment, whereas patients with zero or one factor constituted the lower-risk category. This secondary stratification was used specifically to determine the expected intensity of postoperative management and thereby assess treatment concordance; the intermediate-risk and lower-risk categories were not used as separate groups for comparative survival analyses.

### 2.3. Classification of Guideline Concordance and Potential Overtreatment

Postoperative treatment concordance was evaluated by comparing the treatment actually received with the expected postoperative management according to pathological risk and contemporary guideline recommendations. Within the non-high-risk cohort, the secondary risk assessment described above was used to determine the expected intensity of postoperative treatment.

The 2023 ESGO/ESTRO/ESP recommendations were applied retrospectively as a standardized analytical framework to evaluate postoperative treatment patterns throughout the study period (2012–2025). Accordingly, treatment decisions made before the publication of these recommendations were not considered non-compliant with guidelines that were not yet available at the time of treatment.

For patients in the lower-risk category, observation following radical hysterectomy was considered guideline-concordant management, whereas receipt of postoperative adjuvant treatment was classified as potential overtreatment. For patients meeting the simplified Sedlis-type intermediate-risk definition, observation or guideline-supported adjuvant radiotherapy was considered concordant management. Treatment escalation beyond the expected postoperative management, particularly chemotherapy-containing regimens when no high-risk pathological features were present, was classified as potential overtreatment.

The term potential overtreatment was intentionally used to indicate a postoperative treatment intensity exceeding that expected on the basis of the predefined pathological risk framework and contemporary guideline recommendations. This designation should not be interpreted as evidence that treatment was inappropriate for an individual patient, because treatment decisions may also have been influenced by clinical considerations that were not captured in the retrospective medical records. Similarly, the designation of potential overtreatment does not imply that treatment-related toxicity, impaired quality of life, financial toxicity, or other individual-level harm was demonstrated in this cohort. For operational classification, postoperative management was evaluated using the pathological findings available after radical hysterectomy rather than treatment intensity alone. The pathological risk profile of each patient was first established from tumor diameter, depth of stromal invasion, LVSI, pelvic lymph-node status, parametrial involvement, and surgical margin status. The postoperative treatment actually received was then interpreted in relation to the management expected for that risk profile. Thus, treatment involving multiple modalities was not automatically considered potential overtreatment; classification depended on whether the administered treatment exceeded that supported by the predefined pathological risk category. This approach was intended to distinguish treatment intensity from guideline concordance and to permit a reproducible assessment of real-world postoperative management. Treatment intensity and guideline concordance were therefore treated as conceptually distinct variables: treatment intensity reflected the number of postoperative treatment modalities received, whereas guideline concordance reflected whether the specific treatment administered was supported by the patient’s predefined pathological risk profile.

### 2.4. Study Groups

The principal pathological comparison in the overall cohort involved the high-risk and non-high-risk groups. Patients in the high-risk group were retained to characterize postoperative treatment patterns across the entire study population. However, analyses specifically addressing potential overtreatment and its association with oncologic outcomes were restricted to the non-high-risk cohort. This approach was chosen because treatment escalation, including concurrent chemoradiotherapy, is recommended for patients with high-risk pathological features, whereas excessive treatment intensity can be more meaningfully evaluated among patients without such features [3].

Less intensive postoperative treatment among high-risk patients could potentially represent undertreatment; however, evaluation of undertreatment was outside the primary scope of the present study. Accordingly, the treatment-concordance survival analyses compared guideline-concordant management with potential overtreatment exclusively within the non-high-risk cohort.

### 2.5. Outcomes

The primary outcomes were the proportion of patients receiving guideline-concordant postoperative management and the frequency of potential overtreatment according to the 2023 ESGO/ESTRO/ESP recommendations [3]. Secondary outcomes comprised disease-free survival (DFS), overall survival (OS), postoperative treatment intensity, and the association between pathological risk groups and treatment intensity. Disease-free survival was calculated from the date of primary radical hysterectomy to the first documented disease recurrence or the last follow-up. Overall survival was calculated from the date of primary radical hysterectomy to death from any cause or the last follow-up.

### 2.6. Statistical Analysis

Statistical analyses were conducted using jamovi (version 2.7.30.0; The jamovi project, Sydney, Australia). Categorical variables were reported as counts and percentages and compared with the chi-square test or Fisher’s exact test, as appropriate. Effect size for contingency analyses was quantified with Cramer’s V. Disease-free survival and overall survival were estimated by the Kaplan–Meier method, with survival time measured from the date of primary radical hysterectomy. Survival curves were compared by the log-rank test. Univariable Cox proportional hazards regression was used to estimate hazard ratios (HRs) and corresponding 95% confidence intervals (CIs). All tests were two-sided, with statistical significance defined as *p* < 0.05.

### 2.7. Ethics Statement

The study was approved by the Trakya University Faculty of Medicine Non-Interventional Scientific Research Ethics Committee (Protocol Code: TUTF-GOBAEK 2026/227; Approval No: 09/29; Date of Approval: 4 May 2026). The study was conducted in accordance with the Declaration of Helsinki. Owing to the retrospective nature of the study, the requirement for informed consent was waived by the Ethics Committee.

## 3. Results

### 3.1. Patient Characteristics

**Figure 1:**
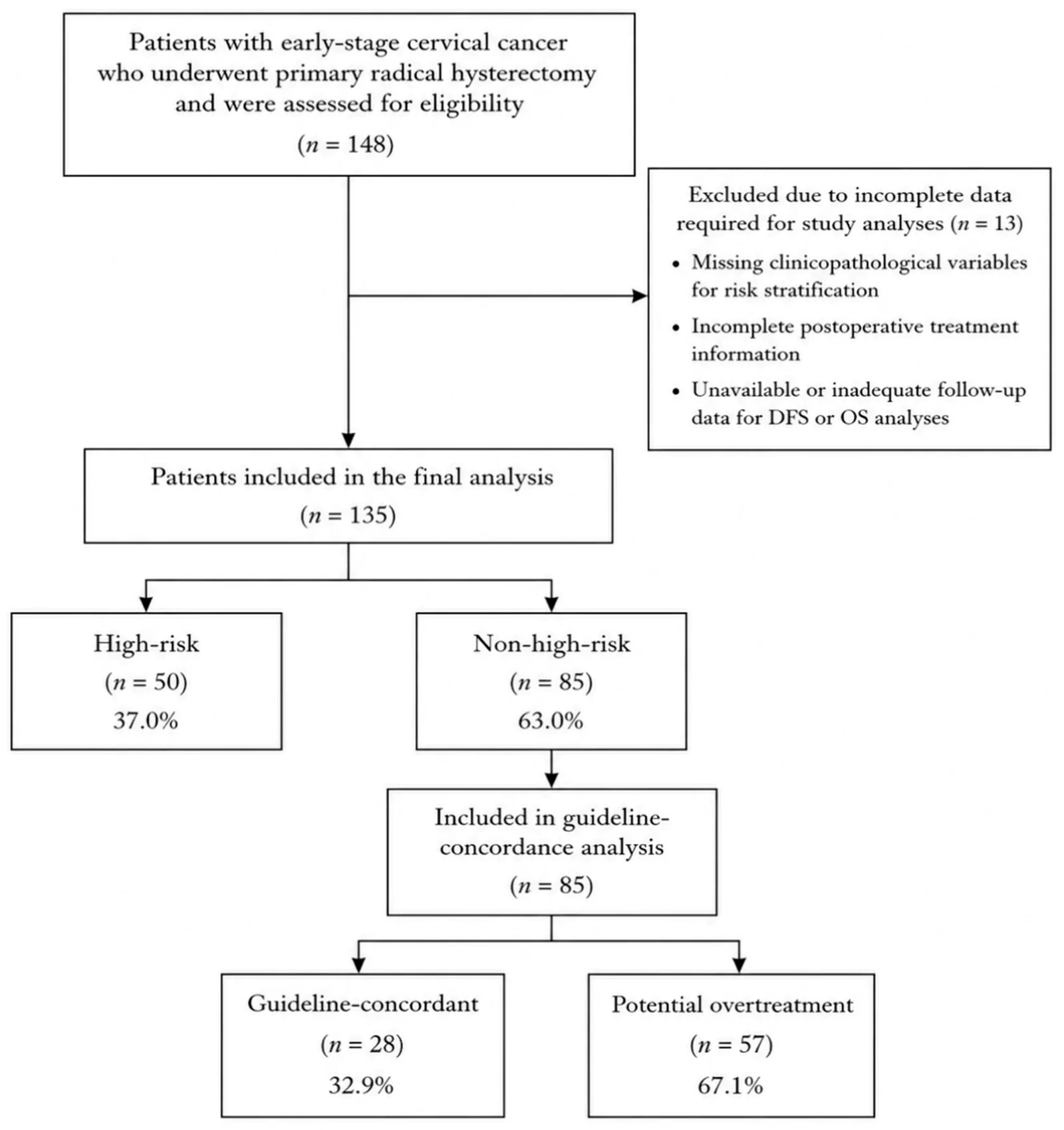
Patient selection and study group classification flowchart.

The final analysis included 135 patients treated with primary radical hysterectomy for early-stage cervical cancer. Median follow-up for the overall cohort was 48 months (IQR, 24–48 months). According to postoperative pathological risk stratification based on the 2023 ESGO/ESTRO/ESP guidelines, 50 patients (37.0%) met the high-risk criteria, while the remaining 85 patients (63.0%) comprised the non-high-risk group. Baseline demographic and clinicopathological characteristics are presented in Table 1, and patient selection and study group classification are illustrated in Figure 1. The non-high-risk cohort (*n* = 85) was subsequently classified according to postoperative treatment concordance for the remaining analyses. Of these patients, 28 (32.9%) received guideline-concordant postoperative management, whereas 57 (67.1%) received treatment classified as potential overtreatment according to the 2023 ESGO/ESTRO/ESP recommendations.

### 3.2. Postoperative Treatment Patterns According to Risk Groups

Postoperative treatment intensity differed significantly by pathological risk group (χ^2^ = 17.8, df = 3, *p* < 0.001; Fisher’s exact *p* < 0.001), with Cramer’s V = 0.363 indicating a moderate effect size (Table 2). Among high-risk patients, multimodal treatment predominated. Only 4.0% of patients received no adjuvant therapy, whereas 20.0% received single-modality treatment, 50.0% received dual-modality treatment, and 26.0% received triple-modality treatment. Treatment intensity among non-high-risk patients was more heterogeneous. Among non-high-risk patients, 27.1% received no adjuvant treatment, 31.8% received single-modality treatment, 28.2% received dual-modality treatment, and 12.9% received triple-modality treatment.

### 3.3. Guideline Concordance and Postoperative Treatment Intensity

Treatment intensity varied significantly according to guideline-concordance status (χ^2^ = 66.6, df = 3, *p* < 0.001; Fisher’s exact *p* < 0.001), with Cramer’s V = 0.885 indicating a very large effect size (Table 3). Within the guideline-concordant group, 82.1% of patients received no adjuvant treatment, whereas 17.9% received single-modality treatment. In contrast, patients who received treatment classified as potential overtreatment frequently received multimodal treatment, including dual-modality therapy (42.1%) and triple-modality therapy (19.3%). Within the non-high-risk cohort, all patients receiving triple-modality treatment met the predefined criteria for potential overtreatment.

### 3.4. Baseline Clinicopathological Characteristics According to Guideline Concordance

To further assess potential baseline differences within the non-high-risk cohort, clinico-pathological characteristics were compared between patients receiving guideline-concordant treatment (*n* = 28) and those receiving treatment classified as potential overtreatment (*n* = 57). No statistically significant differences were observed in age (*p* = 0.722), histological subtype (*p* = 0.689), tumor diameter ≥ 4 cm (*p* = 0.160), deep stromal invasion (*p* = 0.593), LVSI (*p* = 0.658), or FIGO stage distribution (Fisher’s exact *p* = 0.166). Although tumors ≥ 4 cm were numerically more frequent in the potential-overtreatment group (24.6% vs. 10.7%), this difference did not reach statistical significance (Table 4).

### 3.5. Treatment Patterns According to Pathological Risk Category

Among the 85 non-high-risk patients, 21 were classified as intermediate-risk and 64 as lower-risk according to the predefined pathological criteria. Potential overtreatment was observed in 14/21 (66.7%) intermediate-risk patients and 43/64 (67.2%) lower-risk patients, with no significant association between pathological risk category and potential overtreatment (Fisher’s exact *p* = 1.000). Treatment intensity was examined separately from guideline concordance. Among intermediate-risk patients, 9.5%, 33.3%, 33.3%, and 23.8% received no adjuvant, single-modality, dual-modality, and triple-modality treatment, respectively, compared with 32.8%, 31.3%, 26.6%, and 9.4% among lower-risk patients. Although intermediate-risk patients showed a numerical tendency toward greater treatment intensity, the overall distribution did not differ significantly between the two risk groups (Fisher’s exact *p* = 0.092; Pearson χ^2^(3) = 5.99, *p* = 0.112). The distribution of specific postoperative treatment combinations differed significantly between intermediate- and lower-risk patients (Fisher’s exact *p* = 0.006).

### 3.6. Disease-Free Survival

**Figure 2:**
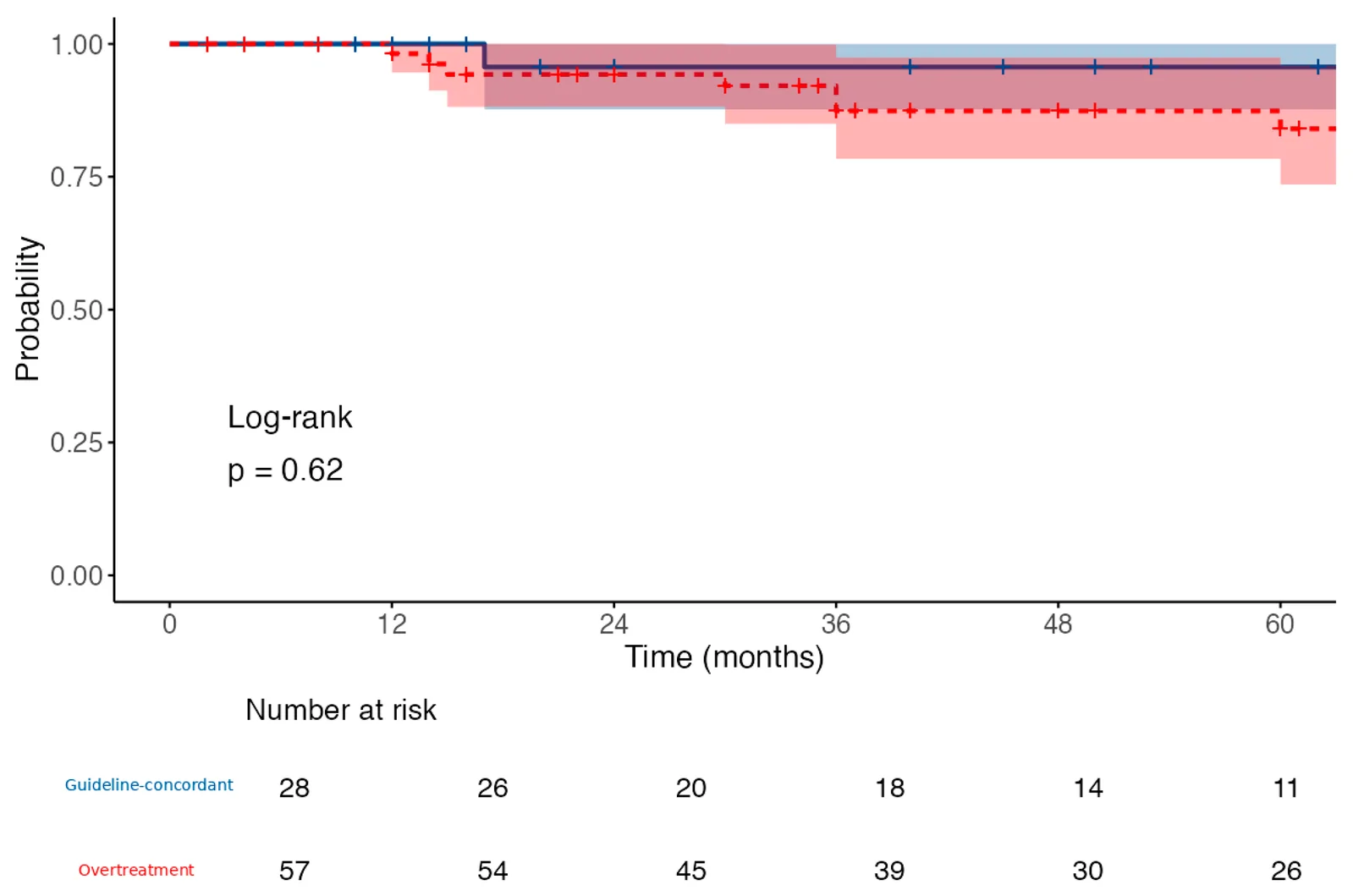
Kaplan–Meier curves for disease-free survival according to guideline-concordance status in the non-high-risk cohort. Tick marks (+) indicate censored observations, and shaded areas represent 95% confidence intervals.

At 5 years, estimated disease-free survival was 95.7% in the guideline-concordant group and 84.0% in the potential overtreatment group. The Kaplan–Meier curves did not indicate a significant difference in disease-free survival between the groups (log-rank *p* = 0.62; Figure 2). In the univariable Cox proportional hazards model, potential overtreatment was not significantly related to disease-free survival (HR = 1.38, 95% CI 0.38–5.06, *p* = 0.624).

### 3.7. Overall Survival

**Figure 3:**
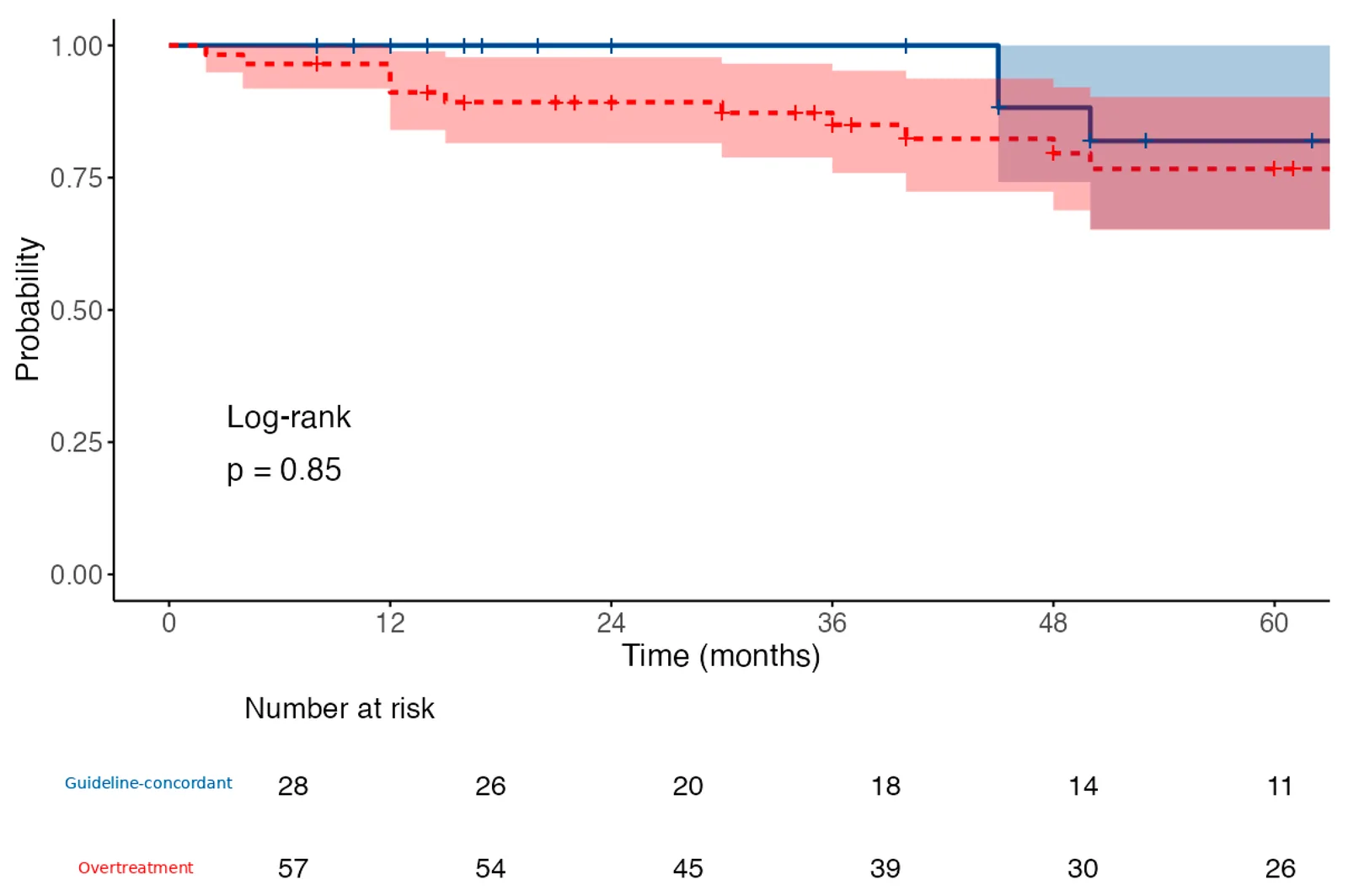
Kaplan–Meier curves for overall survival according to guideline-concordance status in the non-high-risk cohort. Tick marks (+) indicate censored observations, and shaded areas represent 95% confidence intervals.

At 5 years, estimated overall survival was 81.9% in the guideline-concordant group and 76.7% in the potential overtreatment group. The Kaplan–Meier curves did not indicate a significant difference in overall survival between the groups (log-rank *p* = 0.85; Figure 3). In the univariable Cox proportional hazards model, potential overtreatment was not significantly related to overall survival (HR = 1.09, 95% CI 0.42–2.84, *p* = 0.855). The numbers of survival events, estimated 1-, 3-, and 5-year survival probabilities, and univariable Cox regression results for DFS and OS are presented in Table 5.

## 4. Discussion

This retrospective cohort analysis assessed real-world postoperative treatment patterns following primary radical hysterectomy for early-stage cervical cancer in relation to the 2023 ESGO/ESTRO/ESP recommendations, which were applied retrospectively as a standardized analytical framework. Considerable variation in postoperative management was observed among patients without high-risk pathological features. Notably, two-thirds of non-high-risk patients received more intensive adjuvant treatment than expected according to the predefined guideline-based risk framework, and their treatment was therefore classified as potential overtreatment. No statistically significant differences in disease-free or overall survival were observed according to guideline-concordance status; however, the limited number of events and wide confidence intervals preclude exclusion of clinically meaningful treatment effects. Collectively, these findings underscore the importance of evaluating real-world implementation of contemporary guideline recommendations and identifying potentially unnecessary variation in postoperative treatment.

International guidelines advocate risk-adapted postoperative management based on pathological prognostic factors to balance oncologic efficacy against treatment-related toxicity [3,4]. However, translating guideline recommendations into routine clinical practice remains challenging, and variability in guideline-concordant management has been reported across different healthcare settings and institutions [7,8,14]. In the present study, considerable variation in postoperative management was observed among patients without high-risk pathological features, with a substantial proportion receiving treatment exceeding the intensity expected according to the predefined guideline-based risk framework. This variability is likely multifactorial and may reflect institutional practice patterns, multidisciplinary decision-making, and the inherent uncertainty surrounding intermediate-risk disease [14,15,16]. Unlike high-risk disease, where indications for adjuvant treatment are well established, management of intermediate-risk patients requires integration of multiple pathological features rather than reliance on a single definitive criterion, leaving greater room for individualized clinical judgment [3,6]. Accordingly, some degree of variation in postoperative management may persist even in experienced gynecologic oncology centers [15,16]. These findings reinforce the importance of periodically evaluating real-world implementation of contemporary guideline recommendations as part of ongoing quality improvement in gynecologic oncology [15,16].

Potential overtreatment was notably frequent among patients without high-risk pathological features. Importantly, the term “potential overtreatment” was defined strictly according to contemporary guideline recommendations and should not be interpreted as indicating inappropriate clinical decision-making in every individual patient. Rather, it identifies situations in which postoperative treatment exceeded the intensity routinely recommended for the corresponding pathological risk profile. The optimal postoperative management of intermediate-risk disease remains controversial because this population is prognostically heterogeneous and because the available studies have evaluated different questions, including radiotherapy versus observation and chemoradiotherapy versus radiotherapy. Several large multicenter, nationwide, and population-based cohorts have reported no significant survival advantage with routine adjuvant treatment or treatment escalation after adequate radical surgery [10,17,18,19,20]. Similarly, recent systematic reviews and meta-analyses have generally found limited or inconsistent evidence of improved overall survival with more intensive postoperative treatment [9,13,21]. The randomized NRG/GOG-263 trial further demonstrated that adding concurrent cisplatin to postoperative radiotherapy did not significantly improve recurrence-free or overall survival, while substantially increasing severe toxicity and transiently worsening quality of life [12]. Nevertheless, the evidence does not support universal omission of adjuvant treatment. The historical GOG-92 trial and some contemporary observational studies suggest that radiotherapy may reduce recurrence in selected patients, and a recent meta-analysis found a recurrence-free survival benefit with radiotherapy compared with observation, without a corresponding overall survival benefit [22,23]. These apparently divergent findings likely reflect heterogeneity in pathological risk combinations, histology, surgical approach, treatment modality, and patient selection. Therefore, our findings are consistent with a more precise, risk-adapted approach to patient selection and caution against disproportionate multimodal escalation rather than supporting indiscriminate postoperative treatment de-escalation.

Similarly, no statistically significant differences in disease-free or overall survival were observed according to guideline-concordance status; however, these findings should not be interpreted as evidence of therapeutic equivalence. These findings are broadly consistent with recent multicenter, registry-based, and population-based studies reporting no consistent survival advantage from routine adjuvant treatment or treatment escalation after adequate radical surgery in selected intermediate-risk patients [10,17,18,19,20,21]. However, interpretation of the available evidence requires consideration of important differences in study design, patient selection, and treatment comparisons [9,23]. While several contemporary observational studies and systematic reviews have questioned the survival benefit of routine treatment intensification [9,17,18,19,20,21], the randomized NRG/GOG-263 trial specifically examined concurrent cisplatin added to postoperative radiotherapy, rather than adjuvant radiotherapy itself, and did not demonstrate a statistically significant improvement in recurrence-free or overall survival, despite increased treatment-related toxicity [12]. Conversely, the landmark GOG-92 (Sedlis) trial and a recent meta-analysis suggest that postoperative radiotherapy may reduce recurrence in selected intermediate-risk patients, although a consistent overall survival benefit has not been demonstrated [6,23]. These differences may reflect the clinical heterogeneity of intermediate-risk disease, including variations in pathological risk combinations, patient selection, and adjuvant treatment strategies across studies [23,24]. However, these results should be interpreted cautiously. Nonsignificant between-group differences do not establish therapeutic equivalence. Moreover, the retrospective single-center design, limited sample size, and small number of survival events preclude reliably excluding modest treatment effects. Rather than suggesting that adjuvant treatment is unnecessary for all patients without high-risk pathological features, our findings underscore the importance of established pathological risk stratification and contemporary guideline recommendations [3,4], while highlighting the need for prospective randomized and multicenter studies to better identify patients who derive meaningful benefit from treatment intensification [25]. The ongoing CERVANTES trial is expected to provide more definitive evidence by directly comparing observation with adjuvant (chemo)radiotherapy after radical surgery in intermediate-risk disease [24].

Our additional analysis of the non-high-risk cohort further illustrates the complexity of postoperative management in intermediate-risk disease. Although the frequency of potential overtreatment was nearly identical in intermediate- and lower-risk patients (66.7% vs. 67.2%), intermediate-risk patients showed a numerical tendency toward receipt of more intensive postoperative treatment. Importantly, treatment intensity and potential overtreatment represent distinct concepts in this analysis. Treatment intensity describes the number of modalities received, whereas potential overtreatment was determined by whether the specific treatment exceeded that supported by the patient’s pathological risk profile. The significant difference in the distribution of specific postoperative treatment combinations between intermediate- and lower-risk patients further highlights the heterogeneity of real-world management. These findings reinforce the need for risk-adapted rather than intensity-based interpretation of postoperative treatment and highlight the relevance of ongoing prospective studies such as CERVANTES in clarifying optimal management for intermediate-risk disease.

These findings carry several important clinical implications. Current care for early-stage cervical cancer increasingly emphasizes personalized, risk-adapted treatment strategies that optimize long-term oncologic outcomes while preserving quality of life [3,4]. Unnecessary postoperative treatment may expose patients to avoidable gastrointestinal, genitourinary, hematologic, and sexual toxicities [12,26] and may increase treatment burden and contribute to financial toxicity [12,27,28], without a demonstrated additional clinical benefit [12,17]. Beyond treatment selection itself, our findings underscore the importance of systematically evaluating how contemporary guideline recommendations are implemented in routine clinical practice [8,15,16]. Periodic institutional assessment of postoperative treatment patterns may help identify potentially unnecessary variation in care, provide a basis for quality improvement initiatives, and inform efforts to improve consistency in guideline-concordant management [29,30]. Such evaluations may also support multidisciplinary treatment decision-making by providing objective feedback on real-world treatment patterns and their alignment with contemporary evidence-based recommendations [31,32]. Accordingly, rather than supporting indiscriminate treatment escalation, our findings emphasize careful evidence-based, multidisciplinary decision-making tailored to individual pathological risk profiles [12,23,25].

From a clinical quality-improvement perspective, the concordance framework used in this study may provide a practical basis for institutional review of postoperative treatment patterns. Periodic comparison of pathological risk profiles with the adjuvant treatment actually delivered could identify areas in which treatment escalation occurs more frequently than expected and prompt multidisciplinary review of the reasons for such variation. Importantly, this approach should function as an audit and decision-support strategy rather than as a substitute for individualized clinical judgment. When combined with prospective documentation of treatment rationale and patient-level outcomes, guideline-concordance assessment may help institutions monitor changes in practice over time and evaluate the implementation of updated recommendations in routine care.

The relatively high proportion of patients with postoperative high-risk pathological features (37.0%) also warrants consideration from the perspective of preoperative patient selection. In principle, identification of high-risk disease before surgery may influence selection between primary surgical management and definitive chemoradiotherapy and may help avoid subsequent combined-modality treatment. However, the present retrospective dataset did not include sufficiently standardized preoperative clinical and radiological staging information to determine whether the high-risk pathological features identified after surgery could reliably have been predicted preoperatively. Therefore, the proportion of postoperative high-risk cases should not be interpreted as evidence of inadequate preoperative assessment. Nevertheless, these findings underscore the importance of careful preoperative staging and multidisciplinary patient selection, and prospective studies incorporating detailed preoperative imaging and staging data would be required to address this question directly.

Future studies could extend this framework by prospectively documenting the clinical rationale underlying individual postoperative treatment decisions and by evaluating guideline concordance across multiple institutions. Such designs would help distinguish intentional, patient-specific deviations from guidelines from systematic variation in clinical practice. Larger multicenter cohorts would also permit analyses according to individual combinations of intermediate-risk pathological features and specific adjuvant treatment modalities, which could not be reliably examined in the present cohort. Integration of treatment-related toxicity, quality-of-life measures, and patient preferences would further allow future studies to assess whether greater treatment intensity provides a clinically meaningful net benefit beyond conventional survival endpoints.

An important strength of this study is the joint evaluation of retrospective concordance with the 2023 ESGO/ESTRO/ESP recommendations [3] and oncologic outcomes according to guideline-concordance status, with particular emphasis on identifying potential overtreatment among patients without high-risk pathological features [9,13]. To our knowledge, relatively few studies have simultaneously examined postoperative guideline concordance using these contemporary recommendations while also evaluating associated survival outcomes [7,18,19]. Nevertheless, the study has several limitations. First, the retrospective single-center design may restrict the generalizability of our findings to other institutions and healthcare settings [33]. Second, treatment allocation was not randomized and may have been influenced by clinical factors that were not captured within the available medical records [34,35]. Third, the limited number of DFS and OS events reduced statistical precision and resulted in wide confidence intervals; therefore, the study was not adequately powered to exclude clinically meaningful differences in survival between treatment groups. Accordingly, the survival analyses should be regarded as exploratory and interpreted cautiously. Finally, although treatment concordance was evaluated according to the current ESGO/ESTRO/ESP recommendations [3], individual treatment decisions may reasonably have been influenced by patient-specific clinical considerations beyond those included in guideline algorithms. Furthermore, because the study period preceded the publication of the 2023 ESGO/ESTRO/ESP guidelines for a substantial proportion of patients, guideline concordance was assessed retrospectively. Therefore, classifications of potential overtreatment should be interpreted as deviations from the contemporary analytical framework rather than evidence of inappropriate clinical practice at the time of treatment. In addition, treatment-related toxicity, quality-of-life outcomes, and financial toxicity were not systematically available in this retrospective dataset. Consequently, although the study identifies treatment patterns classified as potential overtreatment according to guideline-based recommendations, it cannot directly quantify the clinical or patient-reported harms associated with such treatment.

## 5. Conclusions

In conclusion, considerable variation in postoperative management was observed among patients without high-risk pathological features, with potential overtreatment remaining common in this group. No statistically significant differences in disease-free or overall survival were observed according to guideline-concordance status, although the limited number of events precludes definitive conclusions regarding the oncologic effects of treatment intensification. Continued evaluation of real-world implementation of contemporary guideline recommendations may help identify potentially modifiable variation in postoperative management, thereby facilitating more consistent application of evidence-based risk-adapted treatment strategies and supporting individualized clinical decision-making while minimizing unnecessary treatment-related burden [29,36].

## Figures and Tables

**Table 1:** Baseline demographic and clinicopathological characteristics of the study population.

Non-High Risk (*n* = 85)	High Risk (*n* = 50)	Overall (*n* = 135)	Characteristic
55.6 ± 11.6	57.2 ± 12.3	56.2 ± 11.8	Age, years, mean ± SD
			Histology, *n* (%)
56 (65.9)	39 (78.0)	95 (70.4)	SCC
26 (30.6)	9 (18.0)	35 (25.9)	Adenocarcinoma
3 (3.5)	2 (4.0)	5 (3.7)	Adenosquamous carcinoma
27 (31.8)	37 (74.0)	64 (47.4)	LVSI present, *n* (%)
17 (20.0)	24 (48.0)	41 (30.4)	Tumor diameter ≥ 4 cm, *n* (%)
39 (45.9)	27 (54.0)	66 (48.9)	Deep stromal invasion present, *n* (%)
0 (0.0)	25 (50.0)	25 (18.5)	Pelvic lymph-node metastasis, *n* (%)
0 (0.0)	23 (46.0)	23 (17.0)	Parametrial involvement, *n* (%)
0 (0.0)	17 (34.0)	17 (12.6)	Positive surgical margin, *n* (%)

Continuous variables are reported as mean ± standard deviation (SD), and categorical variables as *n* (%), as appropriate. Percentages are calculated within columns. SCC, squamous cell carcinoma; LVSI, lymphovascular space invasion.

**Table 2:** Distribution of treatment intensity according to pathological risk group.

Treatment Intensity	High Risk (*n* = 50)	Non-High Risk (*n* = 85)	Overall (*n* = 135)
No adjuvant treatment	2 (4.0)	23 (27.1)	25 (18.5)
Single-modality treatment	10 (20.0)	27 (31.8)	37 (27.4)
Dual-modality treatment	25 (50.0)	24 (28.2)	49 (36.3)
Triple-modality treatment	13 (26.0)	11 (12.9)	24 (17.8)
Total	50 (100.0)	85 (100.0)	135 (100.0)

Values are reported as *n* (%), with percentages calculated within each pathological risk group. Treatment intensity was categorized descriptively according to the number of postoperative treatment modalities received (none, single, dual, or triple) and should not be interpreted as equivalent to guideline concordance or potential overtreatment. Guideline concordance was determined separately according to the specific treatment received in relation to each patient’s pathological risk profile. The distribution of treatment intensity differed significantly between pathological risk groups (χ^2^(3) = 17.8, *p* < 0.001; Cramer’s V = 0.363).

**Table 3:** Treatment intensity by guideline concordance.

Guideline Concordance	None	Single	Dual	Triple	Total
Concordant	23 (82.1%)	5 (17.9%)	0 (0.0%)	0 (0.0%)	28 (100.0%)
Potential Overtreatment	0 (0.0%)	22 (38.6%)	24 (42.1%)	11 (19.3%)	57 (100.0%)
Total	23 (27.1%)	27 (31.8%)	24 (28.2%)	11 (12.9%)	85 (100.0%)

Values are presented as *n* (% within guideline-concordance group). Treatment intensity was categorized as none, single-, dual-, or triple-modality treatment. Analysis was restricted to the non-high-risk cohort (*n* = 85).

**Table 4:** Baseline clinicopathological characteristics according to guideline concordance in the non-high-risk cohort.

Characteristic	Guideline-Concordant (*n* = 28)	Potential Overtreatment (*n* = 57)	*p*-Value
Age, years, median (Q1–Q3)	54.5 (48.5–66.5)	53.0 (49.0–64.0)	0.722 ^a^
Histology, *n* (%)			0.689 ^b^
SCC	19 (67.9%)	37 (64.9%)	
Adenocarcinoma	9 (32.1%)	17 (29.8%)	
Adenosquamous carcinoma	0 (0.0%)	3 (5.3%)	
Tumor diameter ≥ 4 cm, *n* (%)	3 (10.7%)	14 (24.6%)	0.160 ^b^
Deep stromal invasion, *n* (%)	14 (50.0%)	25 (43.9%)	0.593 ^c^
LVSI present, *n* (%)	8 (28.6%)	19 (33.3%)	0.658 ^c^
FIGO stage, *n* (%)			0.166 ^b^
IA1	7 (25.0%)	4 (7.0%)	
IA2	0 (0.0%)	2 (3.5%)	
IB1	14 (50.0%)	26 (45.6%)	
IB2	4 (14.3%)	9 (15.8%)	
IB3	0 (0.0%)	5 (8.8%)	
IIA1	1 (3.6%)	7 (12.3%)	
IIA2	2 (7.1%)	4 (7.0%)	

Values are reported as *n* (%) unless otherwise indicated. Q1–Q3, first to third quartile; SCC, squamous cell carcinoma; LVSI, lymphovascular space invasion; FIGO, International Federation of Gynecology and Obstetrics. ^a^ Mann–Whitney U test (U = 760). ^b^ Fisher’s exact test. ^c^ Pearson’s χ^2^ test. Positive surgical margin, pelvic lymph-node metastasis, and parametrial involvement are not presented because the presence of any of these features classified patients as high risk and therefore excluded them from the non-high-risk cohort analyzed here.

**Table 5:** Survival outcomes according to guideline-concordance status.

Outcome	Group	Events, *n*	1-Year, %	3-Year, %	5-Year, %	Cox HR	95% CI	*p*-Value
DFS	Guideline-concordant (*n* = 28)	3	100.0	95.7	95.7	Reference	—	—
	Potential Overtreatment (*n* = 57)	14	98.1	87.4	84.0	1.38	0.38–5.06	0.624
OS	Guideline-concordant (*n* = 28)	6	100.0	100.0	81.9	Reference	—	—
	Potential Overtreatment (*n* = 57)	15	91.1	85.0	76.7	1.09	0.42–2.84	0.855

DFS, disease-free survival; OS, overall survival; HR, hazard ratio; CI, confidence interval. Survival probabilities were obtained from Kaplan–Meier estimates. Cox HRs were derived from univariable Cox proportional hazards models using the guideline-concordant group as the reference. Analyses were limited to the non-high-risk cohort (*n* = 85).

## Data Availability

The data supporting the findings of this study are available from the corresponding author upon reasonable request. The data are not publicly available due to patient privacy and ethical restrictions.

## Abbreviations

The following abbreviations are used in this manuscript:

CI	Confidence Interval
DFS	Disease-free Survival
ESGO	European Society of Gynecological Oncology
ESP	European Society of Pathology
ESTRO	European Society for Radiotherapy and Oncology
HPV	Human Papillomavirus
HR	Hazard Ratio
LVSI	Lymphovascular Space Invasion
NCCN	National Comprehensive Cancer Network
OS	Overall Survival

## References

[1] Bray F., Laversanne M., Sung H., Ferlay J., Siegel R.L., Soerjomataram I., Jemal A. (2024). Global cancer statistics 2022: GLOBOCAN estimates of incidence and mortality worldwide for 36 cancers in 185 countries. CA Cancer J. Clin..

[2] World Health Organization Cervical Cancer. https://www.who.int/news-room/fact-sheets/detail/cervical-cancer.

[3] Cibula D., Raspollini M.R., Planchamp F., Centeno C., Chargari C., Felix A., Fischerová D., Jahnn-Kuch D., Joly F., Kohler C. (2023). ESGO/ESTRO/ESP Guidelines for the management of patients with cervical cancer-Update 2023. Int. J. Gynecol. Cancer.

[4] Abu-Rustum N.R., Campos S.M., Amarnath S., Arend R., Barber E., Bradley K., Brooks R., Chino J., Chon H.S., Crispens M.A. (2025). Cervical Cancer, Version 2.2026, NCCN Clinical Practice Guidelines in Oncology. J. Natl. Compr. Canc. Netw..

[5] Peters W.A., Liu P.Y., Barrett R.J., Stock R.J., Monk B.J., Berek J.S., Souhami L., Grigsby P., Gordon W., Alberts D.S. (2000). Concurrent chemotherapy and pelvic radiation therapy compared with pelvic radiation therapy alone as adjuvant therapy after radical surgery in high-risk early-stage cancer of the cervix. J. Clin. Oncol..

[6] Sedlis A., Bundy B.N., Rotman M.Z., Lentz S.S., Muderspach L.I., Zaino R.J. (1999). A randomized trial of pelvic radiation therapy versus no further therapy in selected patients with stage IB carcinoma of the cervix after radical hysterectomy and pelvic lymphadenectomy: A Gynecologic Oncology Group Study. Gynecol. Oncol..

[7] Chiew K.-L., Chong S., Duggan K.J., Kaadan N., Vinod S.K. (2017). Assessing guideline adherence and patient outcomes in cervical cancer. Asia-Pac. J. Clin. Oncol..

[8] Putri N.Q., Permata T.B.M., Wulandari N.A., Handoko, Nuryadi E., Purwoto G., Gondhowiardjo S.A. (2020). Relationship of adherence to cervical cancer treatment guideline towards patients’ five-year survival: Systematic review of follow-up trials. Cancer Manag. Res..

[9] Ripepi C., Cracco F., Ricci G., Nappi L., Restaino S., Vizzielli G., Carlucci S., Stabile G. (2025). Adjuvant therapy in “intermediate-risk” early-stage cervical cancer: To treat or not to treat? Systematic review and meta-analysis. Cancers.

[10] Cao L., Wen H., Feng Z., Han X., Zhu J., Wu X. (2021). Role of adjuvant therapy after radical hysterectomy in intermediate-risk, early-stage cervical cancer. Int. J. Gynecol. Cancer.

[11] Agustí N., Viveros-Carreño D., Wu C.-F., Wilke R.N., Kanbergs A., Barajas K., Zamorano A.S., Pareja R., Melamed A., Rauh-Hain J.A. (2025). Adjuvant chemoradiotherapy vs radiotherapy alone for patients with intermediate-risk cervical cancer. JAMA Oncol..

[12] Ryu S.Y., Deng W., Albuquerque K., Koh W.J., Mayadev J., Heugel A., Kim B.J., Kim D.Y., Cho C.H., Kim J.W. (2025). Randomized phase III trial of adjuvant radiation versus chemoradiation in intermediate-risk, early-stage cervical cancer following radical hysterectomy and lymphadenectomy: Results from NRG Oncology/GOG-263/KGOG 1008. Ann. Oncol..

[13] Gómez-Hidalgo N.R., Acosta Ú., Rodríguez T.G., Mico S., Verges R., Bebia Conesa V., Bradbury M., Pérez-Hoyos S., Pérez-Benavente A., Gil-Moreno A. (2022). Adjuvant therapy in early-stage cervical cancer after radical hysterectomy: Are we overtreating our patients? A meta-analysis. Clin. Transl. Oncol..

[14] Pfaendler K.S., Chang J., Ziogas A., Bristow R.E., Penner K.R. (2018). Disparities in adherence to National Comprehensive Cancer Network treatment guidelines and survival for stage IB-IIA cervical cancer in California. Obstet. Gynecol..

[15] Boria F., Chiva L., Chacon E., Zanagnolo V., Fagotti A., Kucukmetin A., Mom C., Chakalova G., Shamistan A., Malzoni M. (2022). SUCCOR quality: Validation of ESGO quality indicators for surgical treatment of cervical cancer. Int. J. Gynecol. Cancer.

[16] Ding Y., Zhang X., Qiu J., Zhang J., Hua K. (2022). Assessment of ESGO quality indicators in cervical cancer surgery: A real-world study in a high-volume Chinese hospital. Front. Oncol..

[17] Cibula D., Akilli H., Jarkovsky J., van Lonkhuijzen L., Scambia G., Meydanli M.M., Ortiz D.I., Falconer H., Abu-Rustum N.R., Odetto D. (2023). Role of adjuvant therapy in intermediate-risk cervical cancer patients-Subanalyses of the SCCAN study. Gynecol. Oncol..

[18] Matsuo K., Shimada M., Matsuzaki S., Machida H., Shigeta S., Yoshida H., Kato K., Kanao H., Takekuma M., Mikami M. (2024). Assessment of postoperative therapy de-escalation for early-stage, intermediate-risk cervical cancer. Int. J. Gynecol. Cancer.

[19] Norberg Hardie A., Wenzel H.H.B., Schnack T.H., Van der Aa M.A., Jensen P.T., Bekkers R.L.M., Falconer H., Dutch, Danish and Swedish Gynaecological Oncology (DANDELION) Research Group (2025). Oncological outcomes after radical hysterectomy followed by adjuvant (chemo)radiation or no further treatment in women with cervical cancer FIGO 2018 IB2-IIA2-A multinational population-based cohort study. Eur. J. Cancer.

[20] Baiocchi G., Zanvettor P.H., Lopes A., Ribeiro R., Souza Pinto G.L., Kanaguchi G., Pastore C.B.P., Santos M.H., Melo Filho L.P., Falcao D.F. (2025). Reconsidering adjuvant radiotherapy in intermediate-risk cervical cancer: Findings from the CIRCOL study group. Int. J. Gynecol. Cancer.

[21] da Silva P.H.C.M., Molino G.O.G., Dias M.M.F., Pereira A.G.A., Pimenta N.d.S., Cavalcante D.V.S., de Farias Santos A.C.F., Ferreira S.H., da Silva Santos R., da Silva Reis A.A. (2025). Adjuvant radiotherapy for intermediate-risk early-stage cervical cancer post radical hysterectomy: A systematic review and meta-analysis. J. Clin. Med..

[22] Manso L., Ramchandani-Vaswani A., Romero I., Sánchez-Lorenzo L., Bermejo-Pérez M.J., Estévez-García P., Fariña-Madrid L., García Y.G., Gil-Martin M., Quindós M. (2024). SEOM-GEICO Clinical Guidelines on cervical cancer (2023). Clin. Transl. Oncol..

[23] Yokoi A., Machida H., Okazawa-Sakai M., Nishino K., Nishino H., Itoi M., Mitamura T., Isohashi F., Tsuchida K., Saji H. (2026). Optimal adjuvant strategy in intermediate-risk cervical cancer: A systematic review and meta-analysis. Int. J. Clin. Oncol..

[24] Cibula D., Borčinová M., Kocian R., Feltl D., Argalacsova S., Dvorak P., Fischerová D., Dundr P., Jarkovsky J., Höschlová E. (2022). CERVANTES: An international randomized trial of radical surgery followed by adjuvant (chemo)radiation versus no further treatment in patients with early-stage, intermediate-risk cervical cancer (CEEGOG-CX-05; ENGOT-CX16). Int. J. Gynecol. Cancer.

[25] Taguchi A., Kato K., Hara K., Furusawa A., Nakajima Y., Ishizawa C., Tanikawa M., Sone K., Mori M., Shimada M. (2023). Heterogeneous treatment effects of adjuvant therapy for patients with cervical cancer in the intermediate-risk group. Cancer Med..

[26] Mora-Soto N., Morante-Caicedo C., Caicedo-Martínez M., Viveros-Carreño D., Díaz E.S., Rodríguez J., Vieira S., Peralta J.A., Abu-Rustum N.R., Pareja R. (2025). Morbidity of radical surgery and postoperative radiotherapy in cervical cancer. Int. J. Gynecol. Obstet..

[27] Blanco M., Chen L., Melamed A., Tergas A.I., Khoury-Collado F., Hou J.Y., St. Clair C.M., Ananth C.V., Neugut A.I., Hershman D.L. (2021). Cost of care for the initial management of cervical cancer in women with commercial insurance. Am. J. Obstet. Gynecol..

[28] Bouberhan S., Shea M., Kennedy A., Erlinger A., Stack-Dunnbier H., Buss M.K., Moss L., Nolan K., Awtrey C., Dalrymple J.L. (2019). Financial toxicity in gynecologic oncology. Gynecol. Oncol..

[29] Chargari C., Tanderup K., Planchamp F., Chiva L., Humphrey P., Sturdza A., Tan L.T., van der Steen-Banasik E., Zapardiel I., Nout R.A. (2023). ESGO/ESTRO quality indicators for radiation therapy of cervical cancer. Int. J. Gynecol. Cancer.

[30] Fotopoulou C., Eriksson A.G., Planchamp F., Morice P., Taylor A., Sturdza A., Coza O.F., Halaska M.J., Martinelli F., Armbrust R. (2024). European Society of Gynaecological Oncology expanded quality indicators and accreditation for cervical cancer management. Int. J. Gynecol. Cancer.

[31] Fahim C., McConnell M.M., Wright F.C., Sonnadara R.R., Simunovic M. (2020). Use of the KT-MCC strategy to improve the quality of decision making for multidisciplinary cancer conferences: A pilot study. BMC Health Serv. Res..

[32] Patkar V., Acosta D., Davidson T., Jones A., Fox J., Keshtgar M. (2012). Using computerised decision support to improve compliance of cancer multidisciplinary meetings with evidence-based guidance. BMJ Open.

[33] Kim Y.R., Chung S.W., Kim M.J., Choi W.M., Choi J., Lee D., Lee H.C., Shim J.H. (2024). Limited generalizability of retrospective single-center cohort study in comparison to multicenter cohort study on prognosis of hepatocellular carcinoma. J. Hepatocell. Carcinoma.

[34] Streeter A.J., Lin N.X., Crathorne L., Haasova M., Hyde C., Melzer D., Henley W.E. (2017). Adjusting for unmeasured confounding in nonrandomized longitudinal studies: A methodological review. J. Clin. Epidemiol..

[35] Franklin J.M., Platt R., Dreyer N.A., London A.J., Simon G.E., Watanabe J.H., Horberg M., Hernandez A., Califf R.M. (2022). When can nonrandomized studies support valid inference regarding effectiveness or safety of new medical treatments?. Clin. Pharmacol. Ther..

[36] Vink M.D., Hofstra G., Koolman X., Bekkers R.L., Siebers A.G., van Kemenade F.J., Böcker K.B., ten Hove M., van der Hijden E.J. (2023). Identification of over- and undertreatment in the Dutch national cervical cancer screening program: A data linkage study at the hospital level. Prev. Med. Rep..

